# Une association exceptionnelle d’un liposarcome dédifférencié retro péritonéal et d’un liposarcome bien différencié péri colique: à propos d’un cas

**DOI:** 10.11604/pamj.2016.25.254.10398

**Published:** 2016-12-21

**Authors:** Mohamed Sinaa

**Affiliations:** 1 Service d’Anatomie et de Cytologie Pathologique, Hôpital militaire Moulay Ismail, Meknès, Morocco

**Keywords:** Liposarcome bien différencié, liposarcome dédifférencié, pronostic, Well-differentiated liposarcoma, dedifferentiated liposarcoma, prognosis

## Abstract

Le liposarcome est une tumeur mésenchymateuse maligne de nature adipeuse très rare. L'organisation mondiale de la santé classe les liposarcomes en cinq sous-types: bien différencié, myxoïde, pléomorphe, dédifférencié et mixte. L'association de localisation multiples et synchrones de plusieurs sous types des liposarcomes est exceptionnelle. Seulement 34 cas sont décrits dans la littérature. Nous rapportons une observation d'une association synchrone d'un liposarcome dédifférencié retro péritonéal et deux petits liposarcomes bien différenciés type lipoma-like péri coliques. Les aspects anatomopathologiques, les options thérapeutiques et les facteurs pronostics des liposarcomes seront revus dans cet article.

## Abstract (en)

Liposarcoma is an extremely rare malignant adipose mesenchymal tumor. World Health Organization classifies liposarcomas into five subtypes: well differentiated, myxoid, pleomorphic, dedifferentiated and mixed. The association between multiple and synchronous location of several subtypes of liposarcomas is exceptional. Only 34 cases have been reported in the literature. We report the case of synchronous association between retroperitoneal dedifferentiated liposarcoma and two small well-differentiated lipoma-like pericolic liposarcomas. Anatomopathological aspects, therapeutic options and prognostic factors of liposarcomas have been reviewed in this study.

## Introduction

Le liposarcome est une tumeur maligne des parties molles qui se développe, non pas à partir du tissu adipeux différencié, mais à partir des cellules mésenchymateuses primitives [1, 2]. C'est le plus fréquent des sarcomes des parties molles [1, 2]. Les critères diagnostiques sont bien définis, ainsi que les cinq formes histologiques classées par l'OMS. Le liposarcome est généralement unique et constitué d'une seule variante histologique. Nous rapportons une observation de deux liposarcomes à localisations multiples, rétro péritonéale et péricoliques, synchrones et de types histologiques différents.

## Patient et observation

**Figure 1:**
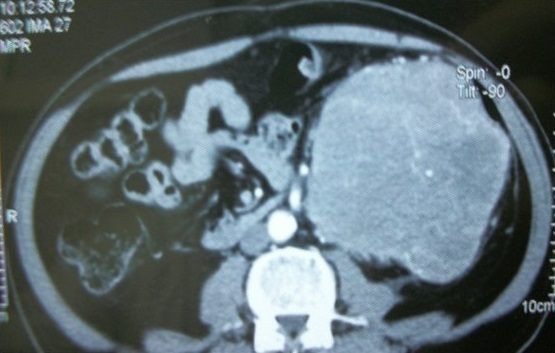
Coupe tomodensitométrique montrant une masse retro péritonéale s’étend vers le flanc gauche

Patient âgé de 68 ans a été hospitalisé pour douleurs abdominales, une augmentation progressive du volume abdominal et d'un amaigrissement non chiffré. L'examen physique a mis en évidence une masse abdominale essentiellement au dépend du flanc gauche. La tomodensitométrie abdominale a révélé une masse rétro péritonéale importante en regard de l'hypochondre gauche qui s'étendu jusqu'à la fosse iliaque gauche (Figure 1), et de deux autres petites masses péricoliques gauche. Une intervention chirurgicale a été décidée et a consisté à la résection d'une masse de la loge rétropéritonéale mesurant environ 28x18x15 cm, ainsi que les deux autres petites masses mesurant successivement 10x8x5cm et 6x4x4cm.

**Figure 2:**
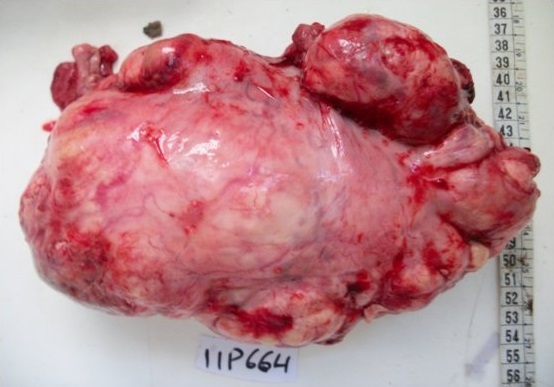
Photographie macroscopique de la masse retro péritonéale: bien limitée, polylobée, avec à la coupe, aspect blanc jaunâtre, fasciculé de consistance variable, myxoïde par place

**Figure 3:**
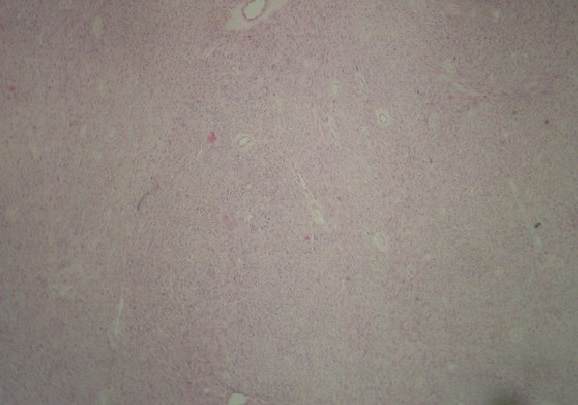
Liposarcome dédifférencié: prolifération sarcomateuse faite de faisceaux tantôt parallèles, tantôt entrecroisé (HEx100)

**Figure 4:**
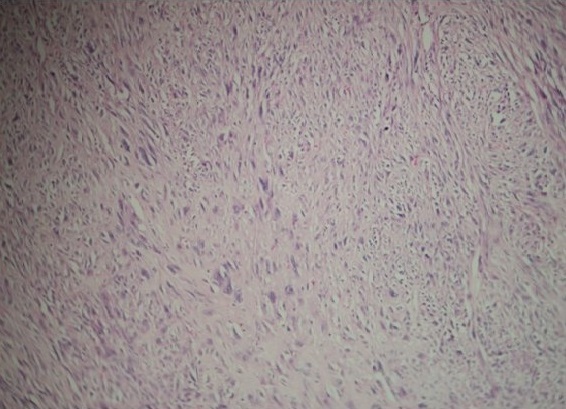
Liposarcome dédifférencié: cellules fusiformes aux noyaux allongés, anisocaryotiques, hyper chromatiques avec des figures mitotiques (HEx200)

**Figure 5:**
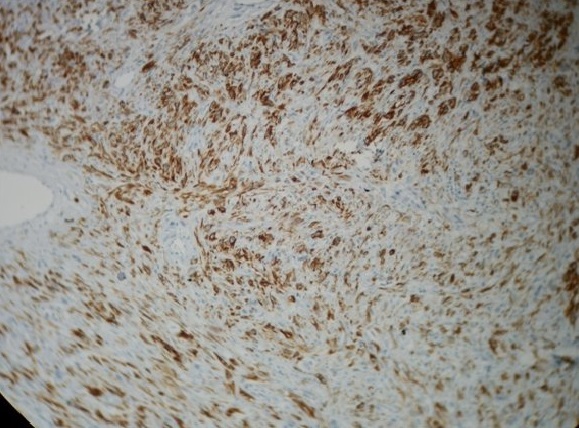
Immunohistochimie: marquage diffuse cytoplasmique par la PS100

**Figure 6:**
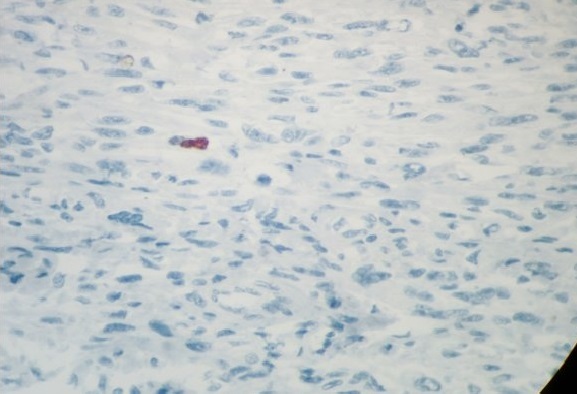
Immunohistochimie: marquage nucléaire focale par le Mdm2

L'examen macroscopique de la masse retro péritonéale notait une tumeur bien limitée polylobé, de coloration blanc jaunâtre et de consistance variable à la coupe avec un aspect fasciculé hétérogène, myxoïde par places sans foyers de nécrose avec zone de remaniements hémorragiques (Figure 2), les deux autres masses ont un aspect graisseux homogène. L'examen histologique de la masse principale trouve une prolifération sarcomateuse indifférenciée faite de faisceaux tantôt parallèles tantôt entrecroisés simulant un fibrosarcome (Figure 3). Les cellules tumorales sont fusiformes, au cytoplasme éosinophile, mal limité, aux noyaux tantôt arrondis, modérément hyper chromatiques, tantôt allongés avec des figures de mitoses (12 mitoses au fort grossissement) sans nécrose ni emboles vasculaires (Figure 4). Il n'est pas vu de signes morphologique de différenciation lipoblastique. L'échantillonnage tumorale a permet de retrouver une composante minime adipeuse bien différenciée associée. Le diagnostic d'un liposarcome a été proposé. Le complément immunohistochimique a été réalisé et a montré une positivité diffuse des cellules tumorales au Desmine et au PS100 (Figure 5) et une positivité multifocale nucléaire au Mdm2 (Figure 6). Par ailleurs, elles sont négatives pour l'Actine muscle lisse, le CD117, et la Myogénine. On a conclu à un liposarcome de type dédifférencié.

**Figure 7:**
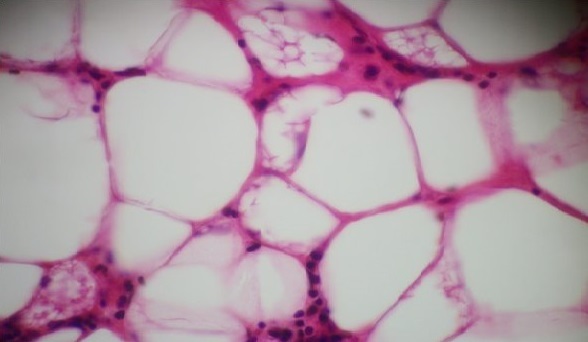
Liposarcome bien différencié lipoma-like: prolifération faite d’adipocytes régulières mêlés à des lipoblastes, au cytoplasme vacuolisé, et aux noyaux hyper chromatiques encochés (HEx 400)

L'examen histologique des deux autres masses péri coliques trouvait le même aspect microscopique. Il s'agit une prolifération tumorale faite de cellules adipeuses bien différenciées formant des lobules séparés par des septas fibreux au sein desquels, on note la présence de cellules de grande taille au cytoplasme multivacuolées, et aux noyaux hyper chromatiques irréguliers parfois encochées ressemblant à des lipoblastes (Figure 7). Ces cellules sont positives pour l'anticorps anti Mdm2. Le diagnostic été en faveur d'un liposarcome bien différencié de type lipoma-like.

Le bilan d'extension a montré la présence de métastases pulmonaires au scanner thoracique. Les suites opératoires étaient simples. Le patient est perdu de vue trois mois après son opération.

## Discussion

Les sarcomes sont des tumeurs mésenchymateuses primitives et rares représentant moins de 1% de l'ensemble des tumeurs malignes [1, 2]. 10 à 15% sont localisés au niveau du rétro péritoine et sont dominés par le liposarcome, et 5% sont localisés au niveau du colon [1–3]. Plusieurs variétés histologiques de malignité croissante ont été décrites: Le liposarcome bien différencié, le liposarcome myxoïde, le liposarcome pléomorphe, le liposarcome mixte et le liposarcome dédifférencié [1, 2].

Le liposarcome dédifférencié rétro péritonéal atteint de façon égale les deux sexes [2]. L'âge moyen au moment du diagnostic se situe vers la cinquième décade, mais la maladie peut intéresser tous les groupes d'âges [2, 3]. Les symptômes révélant la maladie ne sont pas spécifiques. La tumeur est généralement unique [3, 4]. Le liposarcome bien différencié à localisation colique ou péricolique touche essentiellement l'homme avec un âge moyen d'environ 57 ans. Les circonstances de diagnostics sont variables, parfois une découverte fortuite suite à un bilan radiologique (comme c'est le cas chez notre malade) [5]. L'examen histologique de la masse retro péritonéale de notre patient a conclu à un liposarcome de type dédifférencié. Celui des deux autres formations péricoliques a conclu à un liposarcome bien différencié type lipoma-like. Les associations synchrones des liposarcomes rapportées dans la littérature sont généralement constituées du même sous type histologique au sein de la même masse. Les localisations multiples et synchrones de plusieurs types histologiques, tel que le cas chez notre malade, sont exceptionnelles. A notre connaissance, Seulement 34 cas sont décrit dans la littérature [1, 2, 5–7]. Le diagnostic de certitude des liposarcomes est anatomopathologique avec un complément immunohistochimique par l'anticorps anti-Mdm2 et/ou le CDK4 (marquage nucléaire focal ou diffus des cellules lipoblastiques). L'analyse chromosomique ainsi que les études cytogénétiques et moléculaires sont actuellement d'un grand apport pour le diagnostic différentiel de certain tumeurs adipeuses posant un problème de diagnostics. Les liposarcomes bien différenciés sont, quant à eux reconnus par la présence de chromosomes surnuméraires constitués d'une amplification de la séquence 12q14-15 du bras long du chromosome12. L'amplification du gène MDM2 est quasi constante alors que celle des gènes SAS, CDK4 et HMGIC est plus rare [1, 2]. Les liposarcomes dédifférenciés seraient liés à la translocation inverse (12;16) (q13;p11) [1, 2].

Selon la localisation, l'évolution des liposarcomes est longtemps asymptomatique et le diagnostic est souvent tardif. L'exérèse chirurgicale large de la tumeur emportant au besoin les organes de voisinages est la seule alternative thérapeutique de ces lésions, L'intérêt des traitements adjuvants est discutable. La radiothérapie utilisée en pré-ou en post opératoire paraît diminuer le risque de récidives. Étant donnés la faible chimiosensibilité, le bénéfice d'une chimiothérapie est limité [8–11].

Le type histologique de la tumeur représente le principal facteur pronostique. Il est associé aux récidives locales, aux récidives de métastases et à la survie globale [1, 2, 10]. Le liposarcome bien différencié est de meilleur pronostic. Il peut récidiver localement après exérèse, mais il a un pouvoir métastatique faible. La forme myxoïde qui constitue la forme anatomopathologique la plus fréquente (50%) est cliniquement plus maligne, récidive rapidement et est de pronostic plus mauvais. Le liposarcome pléomorphe, le liposarcome mixte ainsi que liposarcome dédifférencié sont de pronostic sombre [1, 2, 10, 11]. D'autres facteurs pronostiques ont été impliqués tels que la résection complète, l'existence de métastases synchrones ainsi que l'atteinte neurovasculaire et osseuse [1, 2, 10].

## Conclusion

Le liposarcome est une entité maligne rare dont le diagnostic de certitude est anatomopathologique. L'association synchrone et de localisation multiples de plus d'un sous type histologique des liposarcomes est exceptionnelle. La résection chirurgicale aussi large que possible constitue le seul moyen thérapeutique.

## References

[1] Coindre JM, Alain Aurias FP (2010). Well-differentiated and dedifferentiated liposarcomas. Virchows Arch. Feb..

[2] WHO (2002). Classification of Tumours of soft Tissue and Bone..

[3] Maàmouri N, Cheikh I, Ouerghi H, Oukaà A, Belkahla N, Mnif E, Hechiche M, Driss M, BenAmmar A (2005). Liposarcome rétropéritonéal géant: à propos d'un cas. La revue de médecine interne..

[4] Granel B, Serratrice J, Andrac-Meyer L, Enea N, Rodriguez F, Bonardel G, Champsaur P, Disdier P, Weiller P-J (2003). Diagnostic difficile d'un liposarcome infraclinique responsable d'une fièvre au long cours: «petite tumeur, grande inflammation ». La revue de médecine interne. Dec..

[5] Lmejjatia M, Loqaa C, Haddia M, Hakkoub M, BenAlia S (2008). Liposarcome primitif du rachis lombaire. Revue du Rhumatisme..

[6] Rais G, Benatiya M, Andaloussi, Raissouni S, Barki A, Allaoui M, Zouaidia F, Afi M, Mrabti H, Errihani H (2011). Liposarcome dédifférencie du cordon spermatique: difficultés thérapeutiques des grosses tumeurs. Pamj..

[7] Antinori A, Antonacci V, Magistrelli P (2002). Giant retroperitoneal liposarcoma. Am J Surg..

[8] Bennani S, Debbagh A, Louahlia S, El Mrini M, Ben Jalloun S (1995). Le liposarcome rétropéritonéal : à propos de deux cas. J Ann Urol (Paris)..

[9] Tsang A, Nash JR, Fordham MV, Hartley MN, Poston GJ (2003). The management of retroperitoneal liposarcoma with synchronous intraduodenal sarcoma. Eur J Surg Oncol..

[10] Dei Tos AP (2000). Liposarcoma: new entities and evolving concepts. Ann Diagn, Pathol..

[11] Schwartz SL, Swierzewski SJ 3rd, Sondak VK, Grossman HB (1995). Liposarcoma dedifferentiated of the spermatic cord: report of 6 cases and review of the literature. J Urol..

